# Collective predictive coding as model of science: formalizing scientific activities towards generative science

**DOI:** 10.1098/rsos.241678

**Published:** 2025-06-04

**Authors:** Tadahiro Taniguchi, Shiro Takagi, Jun Otsuka, Yusuke Hayashi, Hiro Taiyo Hamada

**Affiliations:** ^1^ Graduate School of Informatics, Kyoto University, Kyoto, Japan; ^2^ Research Organization of Science and Technology, Ritsumeikan University, Kyoto, Japan; ^3^ Independent Researcher, Tokyo, Japan; ^4^ Faculty of Social Informatics, ZEN University, Kanagawa, Japan; ^5^ Data Science and AI Innovation Research Promotion Center, Shiga University, Hikone, Japan; ^6^ Center for Advanced Intelligence Projet, RIKEN, Wako, Saitama, Japan; ^7^ AI Alignment Network, Tokyo, Japan; ^8^ ARAYA Inc., Chiyoda-ku, Tokyo, Japan; ^9^ DeSci Tokyo, Tokyo, Japan

**Keywords:** collective predictive coding, model of science, multi-agent system, Bayesian inference

## Abstract

This article proposes a new conceptual framework called *collective predictive coding as a model of science (CPC-MS*) to formalize and understand scientific activities. Building on the idea of CPC originally developed to explain symbol emergence, CPC-MS models science as a decentralized Bayesian inference process carried out by a community of agents. The framework describes how individual scientists’ partial observations and internal representations are integrated through communication and peer review to produce shared external scientific knowledge. Key aspects of scientific practice like experimentation, hypothesis formation, theory development and paradigm shifts are mapped onto components of the probabilistic graphical model. This article discusses how CPC-MS provides insights into issues like social objectivity in science, scientific progress and the potential impacts of artificial intelligence on research. The generative view of science offers a unified way to analyse scientific activities and could inform efforts to automate aspects of the scientific process. Overall, CPC-MS aims to provide an intuitive yet formal model of science as a collective cognitive activity.

## 1. Introduction

Science is a massive cooperative venture of mankind. Even though each person has limited observations of natural phenomena, their own experience, perceptions and actions, we can integrate our knowledge through scientific communication and accumulate scientific knowledge throughout the global human community. Science is a collective behaviour of humans, which explores the world in an active manner. Meanwhile, science has its specific way of communication involving proposing hypotheses, formulating theories, conducting experiments, writing papers, submitting them to journals and conferences, and performing peer reviews. While they have diversity depending on the specific scientific field, most people believe that such scientific activities integrate our knowledge and lead us to better understand the world. However, in what sense do the overall scientific activities bring us a model and better understand the world? How can we improve the rules, regulations, games and habits in our scientific activities? To understand and improve scientific activity itself, a wide range of studies have been undertaken, including philosophy of science; science, technology and society (STS) and science of science [1–5]. Despite these efforts, we have not obtained an intuitive framework that models the total scientific activities. A systematic understanding of science as a whole is also strongly called for in light of recent changes in the scientific landscape, e.g. the introduction of artificial intelligence (AI) technologies [6] and huge problems of research ethics including the replicability crisis [7]. This article proposes a new conceptual and computational framework of science based on the idea of *collective predictive coding* (*CPC*) [8].

The CPC hypothesis was originally proposed to explain the phenomenon of symbol emergence [8–10], namely, the process by which a shared system of symbols, such as language, arises and evolves within a population of agents through their interactions with each other and the environment. The CPC hypothesis extends the idea of *predictive coding* (*PC*) and *free-energy principle (FEP*) [11–15] to a group of people and argues that we, humans, improve prediction capability by updating the symbol system, i.e. external representation systems, we have as a group. In cognitive science and related fields, PC, which can be generalized from a probabilistic viewpoint to the FEP, has become a dominant idea, which regards human cognition as a process of reducing prediction errors [11,16]. The PC and FEP provide a generative view of cognition that takes into account not only perceptual aspects of cognition but also operational aspects, including decision making and active exploration. However, the primary focus of PC and FEP is individual cognitive processes, with little or no attention to how these individual activities are coordinated. In contrast, the CPC hypothesis was proposed as a generative model for symbol emergence, where a shared language emerges as a representational vehicle for coordinating cognitive agents.

**Figure 1.**
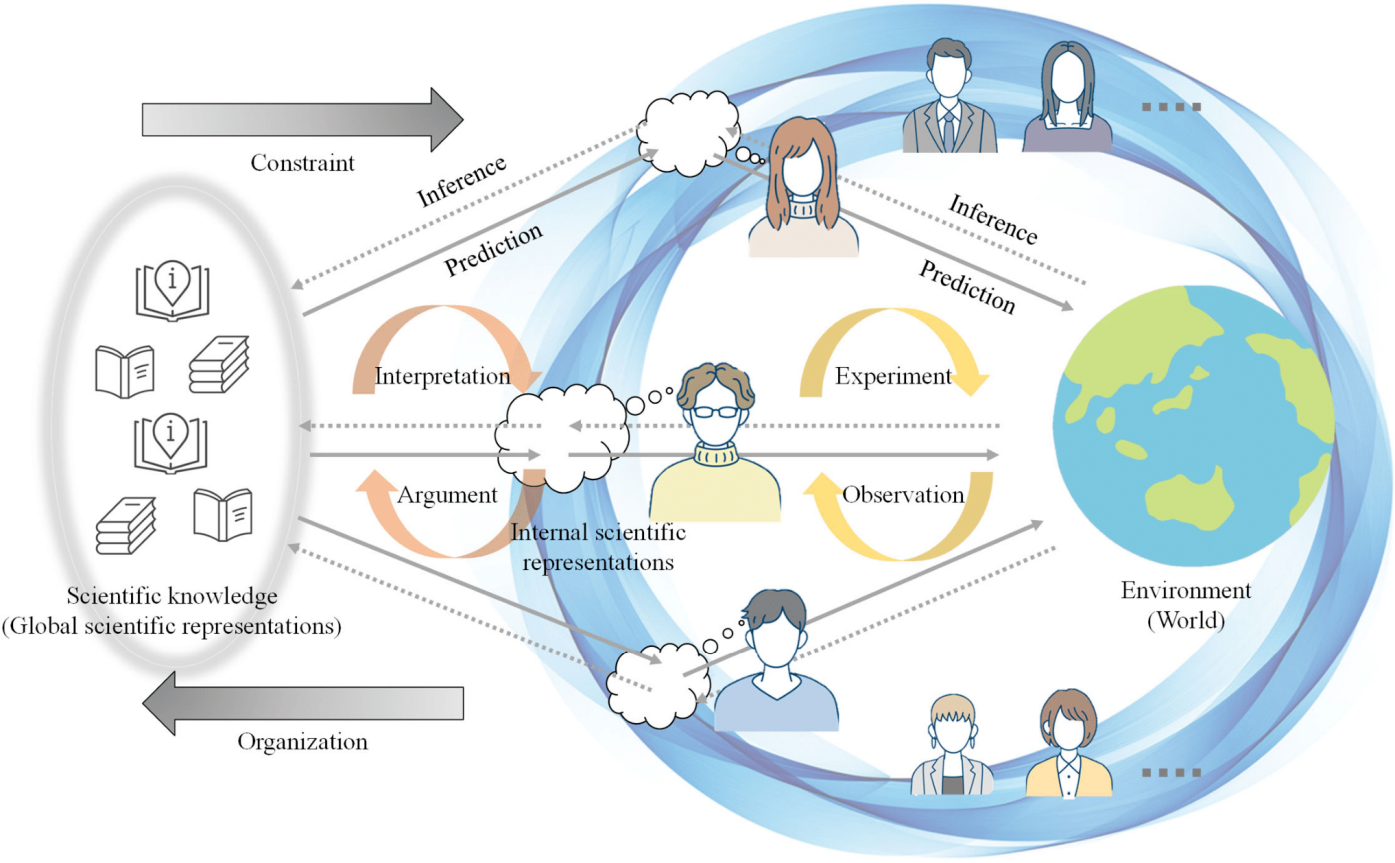
Overview of collective predictive coding as model of science (CPC-MS). This figure illustrates how scientific knowledge emerges through collective processes. Scientists conduct experiments and observations, engaging with the target objects, i.e. environment, through their research methodologies. These individual interactions generate data and insights, which are then synthesized and encoded into a shared system of scientific knowledge, analogous to how language emerges in linguistic systems. The diagram depicts a cyclical nature of this process, where internal scientific activities contribute to and are informed by the collective understanding, forming a dynamic feedback loop between personal research and the broader scientific corpus.

Notably, scientific knowledge we share in our society is also a representative symbol system. This naturally suggests the possibility of extending CPC to an explanatory theory for formalizing science activities (figure 1).

Science—including natural, social and various other disciplines—attempts to model and understand the world. Although the quality of theories in science is evaluated in many ways and the values of theories are not monolithic, prediction capability is a central criterion to evaluate scientific theories [1,3]. This is particularly true in the physical sciences; for example, Newtonian equations describe the generative process that determines the trajectory of a target object [17]. In the context of PC and FEP, robots or AI agents are expected to construct and continuously update an internal model that enables them to predict incoming sensory information and navigate their actions [14,15,18]. Similarly, scientific knowledge accumulated in our society can be seen as an external model that enhances our ability to predict phenomena in the world. In other words, we can regard scientific knowledge as an external representation of an explicit world model, shared and collectively refined by scientists. This view allows us to extend the CPC hypothesis to scientific activities.

Building upon these perspectives, this article aims to introduce a novel conceptual framework called *collective predictive coding as a model of science (CPC-MS*) to formalize and elucidate scientific activities. CPC-MS is a framework that models science as a collective predictive coding activity, as the name suggests. We argue that the general CPC framework extends naturally to encompass various scientific activities, including experimentation, measurement, hypothesis formulation and testing, paper writing, peer review and more. This correspondence between CPC and scientific activities is not conceptually isolated but positioned within a broader research landscape. Prior research has explored variational approaches to niche construction to explain how scientific communities create and maintain shared knowledge structures [19], examined the extension of scientific cognition beyond individual scientists through culturally mediated inferential processes [20], and conceptualized scientific discourse as a form of collective active inference that facilitates adaptive knowledge generation [21].

These complementary perspectives reinforce our approach by demonstrating how the internal/external representation distinction is crucial for understanding collective knowledge systems.

The aforementioned view of science presents a vision to model the entirety of scientific activities as a probabilistic generative model [22]. We term this perspective *generative science*. While developing the concept of generative science in relation to CPC, we establish clear correspondences between the probabilistic generative model underlying CPC and concrete, practical scientific activities. Crucially, CPC-MS posits that shared external representations—scientific knowledge in this context—are updated through a form of peer-review process that functions as a decentralized Bayesian inference [8].

The view of generative *science* proposed in this article also aims to provide a clear view of the total scientific activities from the viewpoint of generative models and allow people who work on science automation to have a more intuitive roadmap for automated science (§4). Since the theory is formulated in terms of probabilistic modelling and machine learning, it offers a natural framework to integrate generative AI into scientific activities. While there has been intense discussion and significant progress in automating individual research tasks, such as information retrieval [23], paper writing [24], hypothesis proposition [6], paper review [25] and experiment execution [26], much work remains to be done in automating the entire social scientific process. CPC-MS may provide a new perspective on such automation.

The remainder of this article is organized as follows. Section 2 introduces CPC as a model for scientific activities, explaining the CPC hypothesis and its application to modelling these activities. This section also includes a detailed explanation of the mathematical framework of CPC-MS, including its formulation based on the theory of active inference; §3 applies the CPC model to understand the social objectivity in science, scientific progress and the shift from confirmatory to generative science; §4 explores the implications of CPC for the future of science, including speculations on AI’s impact on scientific practices and providing guidelines for implementing automated total scientific activity; §5 discusses future research directions; and §6 concludes this study.

## 2. Scientific activities as collective predictive coding

### 2.1. Collective predictive coding hypothesis

**Figure 2.**
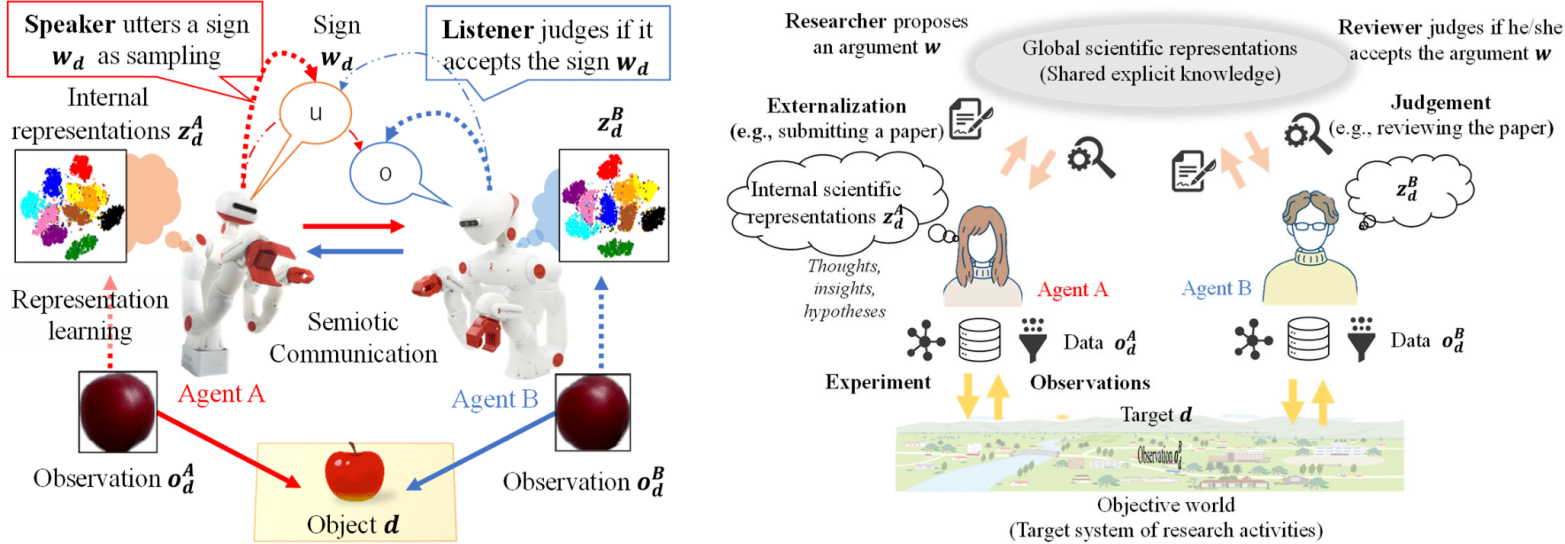
Left: MHNG [27]. In the MHNG, a sign $w_{d}$ representing the dth object is regarded as a sample from an integrated posterior distribution $P(w_{d}|o_{A}^{d},o_{B}^{d})$, where $o_{*}^{d}$ is the ∗-th agent's observation. The naming game acts as a distributed Bayesian inference of the posterior distribution over the shared latent variables $w_{d}$ representing shared external representations. Right: scientific activities updating shared explicit knowledge through experiments and communications involving peer-review process. The total systemic dynamics is structurally analogous to the MHNG.

The CPC hypothesis proposes that the dynamics of human language can be modelled as a process of CPC across society [8]. This hypothesis is based on the mathematical fact that a certain type of language game played among agents, i.e. Metropolis–Hastings naming game (MHNG) (figure 2), can act as a decentralized Bayesian inference of a shared latent variable, sampled instantiation of which can be regarded as an utterance of a name of an object [27]. The CPC hypothesis extended the idea of PC and FEP to the phenomena of language emergence in human society. If the hypothesis is held, human language is considered to collectively encode information about the world as observed by many agents through their sensory–motor systems.

**Figure 3.**
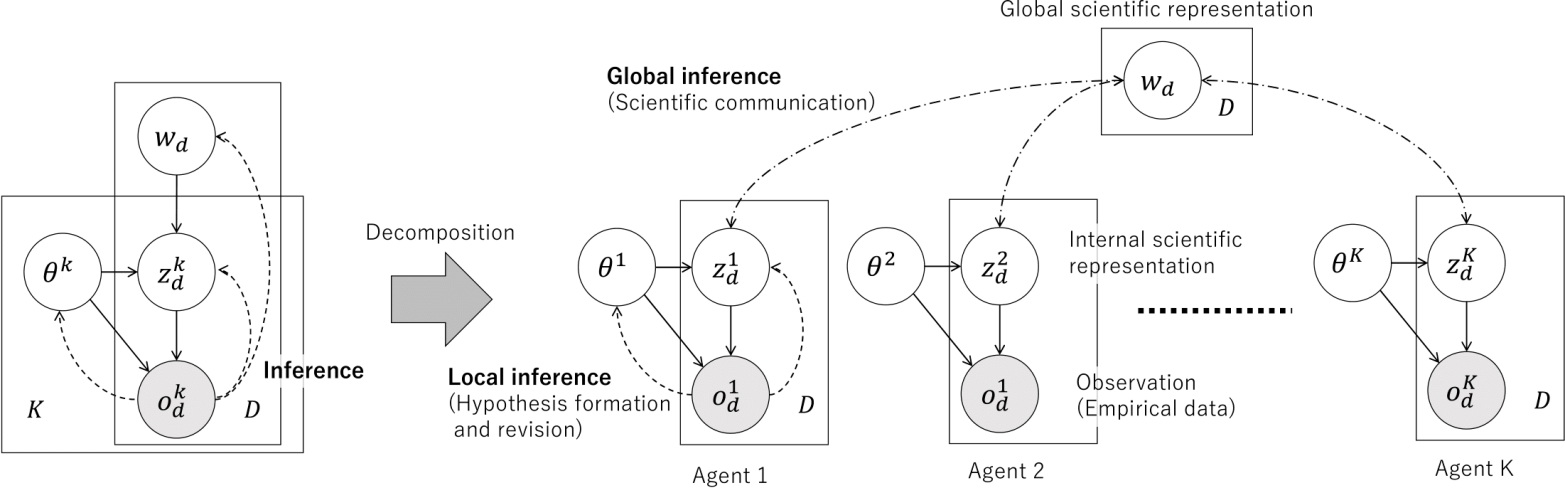
PGM underlying the CPC-MS framework, which is a variant of that of CPC [8]. Left: an integrated model depicting the relationship between individual scientists' observations and a shared global scientific representation. In this framework, the observation $o_{d}^{k}$ of the dth target by the kth scientist is conceptualized as being influenced by the dth global scientific representation $w_{d}$, mediated by the scientist's internal representation $z_{d}^{k}$. The parameter $\theta^{k}$ encapsulates the kth scientist's learned internal models. K and D denote the total number of scientists and targets, respectively. Right: a decomposed version of the model, illustrating how the global scientific representation $w_{d}$ evolves through inter-scientist communication, acting as a decentralized Bayesian inference. This process is exemplified by paper submission and peer review. Through this mechanism, $w_{d}$ emerges as a global scientific representation of the dth target.

From a mathematical perspective, CPC can be modelled using probabilistic graphical models (PGMs) where latent variables representing shared external representations are inferred in a decentralized way through agent interactions and communication. Figure 3 represents a general form of CPC, ignoring the temporal and dynamic aspects of agent–environment sensory–motor interactions, and focusing on representation learning perspectives. The computational model for CPC was obtained by extending the PGM for individual representation learning to a social one. We first defined the total PGM by integrating multiple elemental modules representing agents, i.e. people. Each elemental PGM was assumed to involve latent variables $z_{d}^{k}$ representing the *internal representation* of an agent, and *observations* $o_{d}^{k}$ corresponding to the kth agent. A shared latent variable $w_{d}$ is placed as a parent node of $\{z_{d}^{k}{\}}_{k}$. The latent variable $w_{d}$ represents a sign, e.g. a sentence in language, which can be generally called an *external representation*. The generative model in figure 3 characteristically corresponds to a two-layer hierarchical representation learning system, in which internal representations $\{z_{d}^{k}{\}}_{k}$ owned by each person are influenced by and connected via a prior distribution parametrized by $w_{d}$. Symbol emergence corresponds to the inference of the shared external representation. A nomenclature of variables is provided in appendix A.

The CPC hypothesis assumes that a language game played between agents acts as a decentralized Bayesian inference and the posterior distribution $p(w_{d}|\{o_{d}^{k}{\}}_{k})$ can be inferred approximately. Taniguchi *et al*. proposed MHNG and showed that the naming game can realize the approximate inference of $p(w_{d}|o_{d}^{1},o_{d}^{2})$ in a two-agent setting [27] in a fully decentralized way. Inukai *et al*. extended the idea to N-agent conditions and proposed recursive MHNG [28]. A brief explanation of MHNG is given in appendix B.

Mathematically, the inference of $q\left(z^{k}|o^{k}\right)$ corresponds to representation learning by the kth agent, and $q\left(w|\{z^{k}{\}}_{k}\right)$ is assumed to be estimated through a language game within a group. As a whole, symbol emergence by a group is performed to estimate $q\left(w,\{z^{k}{\}}_{k}|\{o^{k}{\}}_{k}\right)$ in a decentralized manner. We assume that we cannot estimate the true posterior $p\left(w,\{z^{k}{\}}_{k}|\{o^{k}{\}}_{k}\right)$*,* but can only estimate its approximate distribution $q\left(w,\{z^{k}{\}}_{k}|\{o^{k}{\}}_{k}\right)$. In MHNG, q(w|⋅) is considered to be approximated by the sample distribution $q(w|\cdot )\approx \frac{1}{I}\sum_{i}\delta (w,w^{\left[i\right]})$, where $\left\{w^{\left[i\right]}{\}}_{i}∼p(w|\cdot \right)$ and I are the number of samples, i.e. a Monte Carlo approximation.

However, if humans could participate in a language game that enables decentralized inference of $w_{d}$, akin to the MHNG, a symbolic system like language could develop to unify the sensorimotor information collected by individual agents. Consequently, a symbolic system emerges when agents work together in PC. A more detailed formulation from the perspective of the FEP can be found in §2.3.

In summary, the CPC hypothesis proposes that language emerges and evolves as a shared representation system that encodes information about the world in a compressed way, allowing agents to align their models of the world.

### 2.2. Collective predictive coding as a model of science

The main argument of this article is to extend the idea of CPC to a model of science. Assuming that the total goal of science is to build theories that model the world and predict future observations, the framework of CPC seems to be suitable for modelling scientific activities. Especially in science, we need to describe and share theories explicitly, even though insights and thoughts are profoundly internal phenomena in our brains. The explicit theories, which are often described in scientific papers, should be shared among society. We found a clear correspondence between language emergence and scientific activities. We call this framework *CPC as a model of science*, as introduced in §1.

In CPC-MS, it is assumed that each agent has partial observations and limited capability for experimentation and measurement. Based on such partial observations of the world, each agent, or scientist, tries to refine their hypothesis to predict their data more accurately. This corresponds to the representation learning of $z_{d}^{k}$. Based on such a hypothesis, an agent proposes a theory or writes a paper. The paper or theory can be accepted or rejected by reviewers. This is quite similar to the communication in MHNG.

The PGM of CPC (figure 3) provides a framework for understanding and modelling the process of scientific inquiry. The key components of the PGM, including external representations (w), internal representations (z) and observations (o), correspond to different aspects of scientific activities. The parameter θ is a local parameter for the kth agent, which represents agent-specific properties such as the agent’s bias and internal world model.

We assume that scientific activity aims to reveal the structure of the world and encode it into a system of external representations. We consider a scientific paper to represent explicit knowledge that can be interpreted by everyone, unlike tacit knowledge that only the owner can access. Imagine that scientific knowledge consists of a large number of logical descriptions, e.g. mathematical equations of physical laws, structural formulae of compounds in chemistry and descriptive theories of social systems. In this sense, w represents a paper or a theoretical argument. We call w *global scientific representations* in this article.

In contrast, z corresponds to a personal idea about the world. The internal representation z can be explicit or tacit knowledge, including thoughts, insights and hypotheses. We call z *internal scientific representations* in this article.

Table 1 represents the correspondences between mathematical notations in CPC, phenomena in scientific activities and language emergence. Assuming the goal of science is to obtain better external representations, i.e. theories, to better predict the world, within the framework of CPC-MS, CPC of $\{o_{d}^{k}{\}}_{k}$ and Bayesian inference of $w_{d}$ are the goals of science.

**Table 1.** Correspondences between mathematical notations in CPC, phenomena in scientific activities and language emergence.

mathematical notations	science activities	language emergence
external representations w	global scientific representation(e.g. published papers, established theories, consensus models)	shared symbolic system(e.g. words,sentences, signs)
internal representations z	internal scientific representations (e.g. hypotheses, insights, mental models, intuitions)	cognitive representations (e.g. concepts,mental images,perceptual state)
observations o	empirical data (e.g. experimental results, field observations, measurements)	sensory experiences (e.g. visual, auditory, tactile inputs)
inference of P(z|o)	hypothesis formation and revision (e.g. data analysis, theory development)	representation learning (e.g. categorization,concept formation)
inference of P(w|z)	scientific communication (e.g. paper writing, peer review, oral discussion)	language game (e.g. speech production, interpretation)

The following is a scenario of two-agent scientific communication, which can be extended to a multiple-agent setting, playing scientific communication possibly corresponding to a decentralized Bayesian inference in the same way as MHNG. The schematic correspondence between MHNG and the scientific communication is depicted in figure 2.

(1) *Experimentation and measurement*. Each agent (scientist) performs experiments on the dth target and collects observations, which represent the data $o=\{o_{d}^{*}{\}}_{d}$. Due to limited access to the world, scientists can only perform a subset of possible experiments and obtain partial observations.
(2) *Testing and refining hypotheses*. Based on the observations ($o=\{o_{d}^{*}{\}}_{d}$), scientists update their internal scientific representations, i.e. internal representations, ($z_{d}^{*}$) through the inference process P(z|o). This process mirrors how scientists evaluate and refine hypotheses in light of experimental results. Testing hypotheses is represented by the evaluation of the likelihood term P(o|z) in the CPC model, from generative perspectives. Scientists seek hypotheses that maximize the likelihood of the observed data.
(3) *Externalization of scientific representations*. Scientists externalize their ideas mainly by writing papers to communicate their internal representations (z) to the broader scientific community as explicit knowledge. This process can be modelled as a sampling process from the distribution P(w|z), mapping internal representations to external representations (w), which correspond to theories and models described in scientific papers.
(4) *Judgement of scientific representations*. When a scientist submits a paper (w) for review, other scientists evaluate it based on their own internal representations (z). Reviewers assess the compatibility of the proposed theory with their understanding and their beliefs. If successful, the distribution of accepted papers can be regarded as samples of $q(w|\{z^{k}{\}}_{k})$, similar to the MHNG. In other words, when reviewer $k^{\prime }$ makes acceptance decisions based on $p(z^{k^{\prime }}|w)$, the sample distribution of w is influenced by reviewer $k^{\prime }$’s knowledge, resulting in a sample distribution that approximates $p(w|z^{k},z^{k^{\prime }})$ rather than $p(w|z^{k})$. This means that through the judgement process, reviewer $k^{\prime }$’s knowledge is integrated into the distribution.^1^ In other words, this scientific communication consisting of sampling, i.e. externalization, and judgement, i.e. peer review process, is considered as the approximate (decentralized) Bayesian inference of P(w|z) in the CPC framework.
(5) *Iteration*. The process returns to step 1, with scientists designing new experiments based on the updated consensus, continuing the cycle of scientific inquiry.

Through ongoing communication of theories and evidence via papers and discussions, the scientific community, here the group of two agents, updates its shared understanding of the world. This process corresponds to the decentralized Bayesian inference of shared external representations (P(w|o)). In generative science, the ideal scientific consensus corresponds to the inference of the posterior distribution, i.e. $p\left(w|\{o^{k}{\}}_{k}\right)\approx q\left(w|\{o^{k}{\}}_{k}\right)$. Notably, the CPC-MS framework models social scientific activity from the viewpoint of Bayesian inference that aggregates the evidence obtained by agents who have partial capabilities. The CPC-MS framework does not necessarily provide a method enabling the agents to reach the objective ‘truth’, but rather provides a view of scientific communication to better understand, justify and refine the mechanism of scientific activities played by the multi-agent system, i.e. scientists.

By mapping the components of the CPC framework to specific scientific activities, we can see how the PGM provides a unified view of the scientific process. The interplay between observations, internal representations and external representations in the CPC model mirrors the way scientists gather evidence, propose hypotheses, test theories and communicate their findings to the broader community. The CPC framework offers a principled way to understand the emergence of scientific knowledge through the collective efforts of individual scientists.

### 2.3. Active inference on collective predictive coding as a model of science

In transitioning from a MHNG-based perspective of the CPC model to one grounded in active inference, we open a new dimension of understanding scientific processes. Active inference [29], which has been pivotal in explaining cognitive processes through the minimization of prediction errors, offers a robust framework for reinterpreting the CPC model. This approach not only aligns with the principles of active inference but also provides a comprehensive view of scientific inquiry as a collective cognitive process. By adopting this perspective, we can better elucidate how individual scientists’ observations and hypotheses are integrated into a cohesive body of scientific knowledge, facilitating a deeper understanding of the dynamics of scientific progress and the role of collective intelligence in scientific discovery. This shift in perspective underscores the potential of CPC-MS to model science as an active, generative process, driven by the continuous interplay between prediction and observation within the scientific community [8,30].

To operationalize this active inference perspective within CPC-MS, we must consider how scientific communities overcome information asymmetry among individual scientists. Recent research has explored various approaches to collective inference processes among agents with asymmetric information. One approach based on the concept of ‘federated inference and belief sharing’ proposes a model for how agents with different observation spaces can still perform collective inference effectively [31]. While such research focuses on belief integration, our CPC-MS framework takes a complementary approach by emphasizing the emergence of shared external representations (scientific knowledge) through communication protocols analogous to the MHNG. Both existing approaches and CPC-MS address the fundamental challenge of information asymmetry in multi-agent systems, but differ in their mechanisms for consensus formation. Federation-based models primarily focus on how individual agents update their beliefs by incorporating others’ inferences, whereas CPC-MS additionally models the emergence of explicit external representations that serve as communal scientific knowledge. This distinction provides the foundation for our mathematical formulation of CPC-MS as a generative model that captures both individual cognitive processes and collective knowledge creation.

Because the CPC-MS regard the science community as a collective intelligence performing a decentralized Bayesian inference from the viewpoint of generative models, the system as a whole is also viewed as an integrated cognitive system that performs free-energy minimization and active inference [32]. Within the system, each individual is driven to explore based on their individual and collective motivations. Active inference is a theory that explains how living things understand their surroundings, act in the world and learn. It is based on the idea that organisms try to minimize the difference between what they expect and what they actually experience, both now and in the future. Several studies have demonstrated that active inference can explain how organisms balance exploring new options with exploiting known rewards. This approach offers a clear mathematical solution to a long-standing problem in decision-making research.

Mathematically, the CPC-MS in the simplest case can be described as follows:


(2.1)Generative model:p(w,z,o∣a,C)=p(w)p(o∣z,a,C)p(z∣w,a),(2.2)Inference model:q(w,z,o∣a,C)=q(w∣z)q(o∣C)q(z∣o,a),

where w is external (collective) representations, $\text{z}=\{z^{k}{\}}_{k}$ is the set of representations by the kth agent, $\text{o}=\{o^{k}{\}}_{k}$ is the set of observations by the kth agent, $\text{a}=\{a^{k}{\}}_{k}$ is the set of sequence of actions taken by the kth agent and $\text{C}=\{C^{k}{\}}_{k}$ is the set of sequence of rewards taken by the kth agent. The inference of q(z∣o,a) corresponds to representation learning by the kth agent, and q(w∣z) is assumed to be estimated through a language game within a group. As a whole, symbol emergence by a group is performed to estimate q(w,z∣o,a,C) in a decentralized manner. We assume that we cannot estimate the true posterior p(w,z,o∣a,C)*,* but can only estimate its approximate distribution q(w,z∣o,a,C).

Based on these premises, the negative variational free energy—*F—*of the CPC-MS, which exactly corresponds to the model’s variational lower bound (ELBO), is defined as follows:


(2.3)F=DKL[q(w,z,o∣a,C)‖p(w,z,o∣a,C)](2.4)=∫q(w,z,o∣a,C)ln⁡q(w,z,o∣a,C)p(w,z,o∣a,C) dwdzdo(2.5)=−Eq[ln⁡p(w)q(w∣z)]−Eq[ln⁡p(o∣z,a,C)q(o∣C)]−Eq[ln⁡p(z∣w,a)q(z∣o,a)](2.6)≃Eq[ln⁡q(w∣z)]⏟Collective term−Eq[ln⁡p(o∣z,a,C)]−Eq[ln⁡p(z∣w,a)q(z∣o,a)]⏟Individual term(2.7)=Eq[ln⁡q(w∣{zk}k)]⏟Collective regularization−∑kEq[ln⁡p(ok∣zk,ak,Ck)]⏟Individual prediction error−∑kEq[ln⁡p(zk∣w,ak)q(zk∣ok,ak)]⏟Individual regularization,

where the first term $E_{q}\left[\ln q\left(w|\text{z}\right)\right]$ represents the collective regularization, which corresponds to the maximization of the log-likelihood concerning the collective latent variable w; the second term $-E_{q}\left[\ln p\left(\text{o}|\text{z},\text{a},\text{C}\right)\right]$ represents the sum of the prediction error for each kth agent concerning their observation $o^{k}$; and the third term $-E_{q}\left[\ln\;\frac{p\left(z|w,a\right)}{q\left(z|o,a\right)}\right]$ represents the sum of the regularization for each kth agent, indicating how useful the generation of the latent variable $z^{k}$ is for estimating the environmental system for each kth agent. It is important to note that $E_{q}\left[\ln\;p\left(w\right)\right]$ and $E_{q}\left[\ln q\left(\text{o}|\text{C}\right)\right]$ are constant terms and therefore do not affect the parameter estimation in the inference model. Additionally, while individual term can be described as the sum of the values for each kth agent, collective term cannot be expressed as a sum over k.

In equation (2.7) , the *individual prediction error* term represents an individual’s predictive capability based on their internal scientific representations ${\left\{z^{k}\right\}}_{k}$. In generative science, we take the stance of evaluating theories and hypotheses based on how well they can predict observed values, and this term corresponds to that. The *individual regularization* term signifies how consistent the inferred $z^{k}$ based on the external and explicit scientific knowledge w is with the inferred $z^{k}$ based on one’s own observation $o^{k}$. This term represents the interaction between top-down and bottom-up information, which is essential for PC and FEP. While the above two terms appear in general FEP, the first term, the *collective regularization* term, is essential for CPC. The existence of this term gives rise to language and scientific knowledge as external representation systems. This term suggests that it is better for the w represented or inferred by all agents to be as consistent as possible. Notably, both $z^{k}$ and w do not come from the environment but are created by people. The collective regularization term works to adjust latent variables. This means that there is arbitrariness here. In other words, the symbol w representing $z^{k}$ inferred via $q(z^{k}|o^{k},a^{k})$ has various degrees of freedom as long as it satisfies social consensus represented by the collective regularization term. This corresponds to the ‘arbitrariness’, which is considered to be a nature of symbols in semiotics [33]. We, humans, are adapting to the environment as a group and modelling the world in a generative manner, not only through synaptic plasticity in our nervous system but also by using semantically and structurally flexible symbol systems in society with this plasticity. CPC-MS expresses this quality of scientific theories that enables the group to be an adaptive predictor, i.e. a group of agents attempting to model the world.

The expected free energy $G\left(\tilde{\text{a}}\right)$ of the CPC-MS for future observational data is defined as follows:


(2.8)G(a~)=Eq(w~,z~,o~∣a~,C~)[F~](2.9)=∫q(w~,z~,o~∣a~,C~)F~ dw~ dz~ do~(2.10)=Eq[ln⁡q(w~∣{z~k}k)]⏟Collective epistemic value −∑kEq[ln⁡p(o~k∣z~k,a~k,C~k)]⏟Individual pragmatic value −∑kEq[ln⁡p(z~k∣w~,a~k)q(z~k∣o~k,a~k)]⏟Individual epistemic value .

Scientific progress is exploratory in nature. The CPC theory provides a new perspective on collective exploration in science. Individual scientists explore research fields and reach scientific discoveries through hypothesis generation, experimentation, simulation, etc. Drivers of human exploration including scientific activities can be based on extrinsic and intrinsic motivations [34–36]. Intrinsic motivation of theme selection and scientific discovery is especially described as individual drivers such as curiosity. This individual exploration of science can be supported by theories of information gain. The information gain theory states that the relationship between curiosity and confidence is a inverted U-shape function [37]. Information gain as a proxy of curiosity measures the reduction of uncertainties after samplings. This information-theoretic principle inspired multiple related studies on agents’ exploration [38–40].

Analogous to the information gain theory, individual scientists attempt to seek novel scientific discovery by rewarding gaining new information based on current situation. The information gain theory of curiosity states that exploratory behaviours are driven by information gain between what an agent knows (internal representation $z^{k}$) and what an agent obtains (observation $o^{k},a^{k}$). This process can be formulated as refinement of a hypothesis $q(z^{k}|o^{k},a^{k})$. However, this view lacks organizational aspects of science since theme selection of scientists is constrained by multiple collective factors such as scientific trends, grant opportunities and collaborators. In other words, individual exploration of science can be biased by global constraints. These global constraints may be interpreted as external (collective) representation w.

The CPC theory can capture collective aspects of scientific discovery and exploration as hypothesis updating and hypothesis testing. We here describe different exploratory patterns and scientific activities.

— *Hypothesis test.* In scientific activities, doing an experiment is crucial for testing hypothesis. Hypothesis testing can be driven by sampling based on researcher’s internal model and theories as global representation.
— *Hypothesis update*. Updating a hypothesis is another important scientific activity. Updating an internal model of a researcher as hypothesis update is driven by the information gain between models given by observation and global representation.
— *Theme selection*. Theme selection is also exploration in scientific activities. This exploration can be driven by comparisons between information gains from multiple targets $IG_{d_{1},d_{2},...,d_{m}}$. Each scientist has preference on which target is more interesting or curious to him or her. Such preference to hypothesis update promotes theme selection of scientific topics in a decentralized manner.

In summary, the CPC theory can describe multiple collective aspects of scientific discovery such as hypothesis test, hypothesis update and theme selection.

### 2.4. Example

For illustrative purpose, let us explain the case of ‘verifying whether drug X has a pharmacological effect on disease Y’ within the CPC-MS framework.

(1) *Experimentation and measurement*. A research team k∈K designs and conducts an experiment to investigate the effect of drug X on disease Y. For example, they perform clinical trials and collect data $o_{d}^{k}$ on patients’ symptom improvement and biomarker changes. The action $a_{d}^{k}$ here corresponds to the specific experimental procedures, such as administering the drug, measuring biomarkers and recording patient outcomes. Though multiple research teams can obtain their respective $o_{d}^{k}$(k∈K), each group can only perform a subset of possible experiments and obtain partial observations due to ethical constraints, experimental design limitations and limitations, such as constraints on experimental equipment and number of subjects.
(2) *Testing and refining hypotheses*. Research team k analyses the observational data $o=\{o_{d}^{k}{\}}_{d}$ and evaluates whether drug X shows a statistically significant effect on disease Y. Based on this, they update their hypothesis (internal representation) $z_{d}^{k}$ about the effect of drug X. This process is represented as approximate inference of the posterior distribution p(z|w,a). For instance, they refine their hypothesis by evaluating the presence and strength of drug X’s effect through statistical analysis. From a generative perspective, the fitness of hypothesis to the data corresponds to evaluating the likelihood term p(o|z,a,C). The reward $C^{k}$ in this context could represent the potential benefits of the drug, such as improved patient outcomes or reduced healthcare costs, which motivate the research. CPC-MS assumes that researchers pursue hypotheses that increase the likelihood of the observational data while being influenced by the top-down effect p(z|w,a) (prior distribution) given by w, which represents prior knowledge in the field. This aligns with Bayesian inference as p(z,o|w,a,C)∝p(z|w,a)p(o|z,a,C), appropriately updating one’s hypothesis under the influence of prior knowledge w. If the hypothesis (z) is supported, they may plan additional experiments to obtain more data (o) for further verification. If not supported, they consider the reasons and either review the experimental methods to verify the hypothesis or modify the hypothesis (z).
(3) *Externalization of scientific representations*. Researchers externalize their internal scientific representations z as papers and communicate them to the scientific community. This can be modelled as a sampling process from the distribution P(w|z). Specifically, they write a paper w on the theory and data analysis results regarding the effect of drug X to convert internal representations into external representations.
(4) *Judgement of scientific representations.* When a paper w is submitted, other researchers (i.e. reviewers) evaluate it. Reviewers judge the validity of the proposed theory based on their internal representations z, which are informed by existing knowledge in the world and their own expertise. They evaluate aspects such as the appropriateness of the experimental design, the accuracy of statistical analysis and the validity of conclusions.
  This corresponds to the listener’s acceptance judgement in MHNG, and when done properly, the distribution of accepted papers can be viewed as samples from $q(w|\{o_{d}^{k}{\}}_{k})$. The scientific activities through this process, i.e. CPC-MS, can be considered as approximate distributed Bayesian inference of $p(w|\{o_{d}^{k}{\}}_{k}))$.
(5) *Iteration*. The process returns to step 1. Based on the updated scientific consensus, researchers design new experiments. Now, other research teams $k^{\prime }$ conduct experiments on the same target d. For example, they plan experiments to verify the effect of drug *X* in different patient groups or to elucidate the detailed mechanism of action, continuing the cycle of scientific inquiry.

In this way, CPC-MS represents scientific inquiry not so much as ‘the pursuit of true descriptive knowledge $w^{*}$’ but rather as ‘an overall mechanism for integrating knowledge from limited observations’

## 3. Explaining the scientific activities with the collective predictive coding as a model of science

This section examines the CPC-MS framework’s implications for understanding the social dynamics and progress of scientific inquiry. By emphasizing the collective nature of knowledge formation, it challenges the traditional view of science as an individual, confirmatory process. Instead, the framework highlights how diversity and social interactions among scientists contribute to objective knowledge and drive scientific advancement. Additionally, the Bayesian perspective reframes scientific progress not as the accumulation of truths but as the continuous refinement and generation of new ideas, emphasizing the generative aspects of scientific activity.

### 3.1. Social objectivity

One salient feature of the CPC-MS is that scientific knowledge emerges through social interactions among individual scientists who have only partial and possibly distorted understanding of the world. Importantly, the establishment of a global scientific understanding does not entail the consensus among individual scientists: they may well disagree with each other ($q(z^{k}|o)\neq q(z^{l}|o)$ for k≠l) or have incorrect posterior ($q(z^{k}|o)\neq p(z^{k}|o)$) even at the limit where the community as a whole approximately attains the true posterior $q\left(w|\{o^{k}{\}}_{k}\right)\approx p\left(w|\{o^{k}{\}}_{k}\right)$. The holders of scientific knowledge, therefore, are the scientific community as a whole rather than individual scientists. This aligns with the social approach to scientific knowledge [41,42], which frames the objectivity of scientific inquiry as arising from the interplay of social interactions and empirical investigation. In this picture, scientists are depicted not as detached truth seekers who strictly adhere to universal methodologies, but as situated individuals influenced by their backgrounds, beliefs and values. The individual biases, however, get corrected in critical dialogues including peer reviews and replications of experimental results.

The CPC-MS provides a Bayesian model for such social processes through which objective knowledge emerges from diversity. As described in §2, scientists in the CPC-MS model may have their own parameter values θ and thus form different inner representation z based on the same observation o. Thus, their research output w well reflects individual biases. This result, however, will not be integrated into the scientific body unless it is accepted by other scientists (i.e. reviewers), who make the decision on the basis of their own probability criterion q(w|z). The key observation of the CPC-MS model is that such mutual criticisms can be seen as an essential part of the decentralized Bayesian inference, e.g. MHNG, which realizes the inference to the posterior distribution at the scientific community as a whole.

Moreover, our model predicts the ways in which diversity and heterogeneity even promote objective scientific investigations, by allowing for the inclusion of diverse perspectives and the exploration of varied research paths. First of all, Bayesian inference becomes skewed or biased if data concentrate on a limited range, e.g. if the scientific community conducts experiments and observations only within a specific range of interests and subjects. Second, the asymptotic convergence to the true posterior via the repeated decentralized sampling is conditioned by the sampling process being *ergodic*, which roughly means that every part of the hypothesis space is eventually explored. This condition is violated if, among others, there is any ‘blind spot’ theory w that has no positive probability of being proposed by any scientist (so that the Markov chain becomes *reducible*), or if scientific activities are trapped in cycles, with no chance of escaping and exploring new theoretical possibilities (so that the chain is *periodic*). Moreover, an effective convergence of Metropolis–Hastings algorithm requires that samples are independent of each other, which, in the present context, is translated as that scientists carry out their research according to their own interests and concerns. In contrast, if particular topics or groups become too influential within a research community, scientific explorations can become skewed towards a specific region of the hypothesis space, resulting in slower convergence. These pitfalls may be avoided by increasing the diversity of a scientific community, ensuring a wide range of perspectives and approaches are represented.

### 3.2. Scientific progress

**Figure 4.**
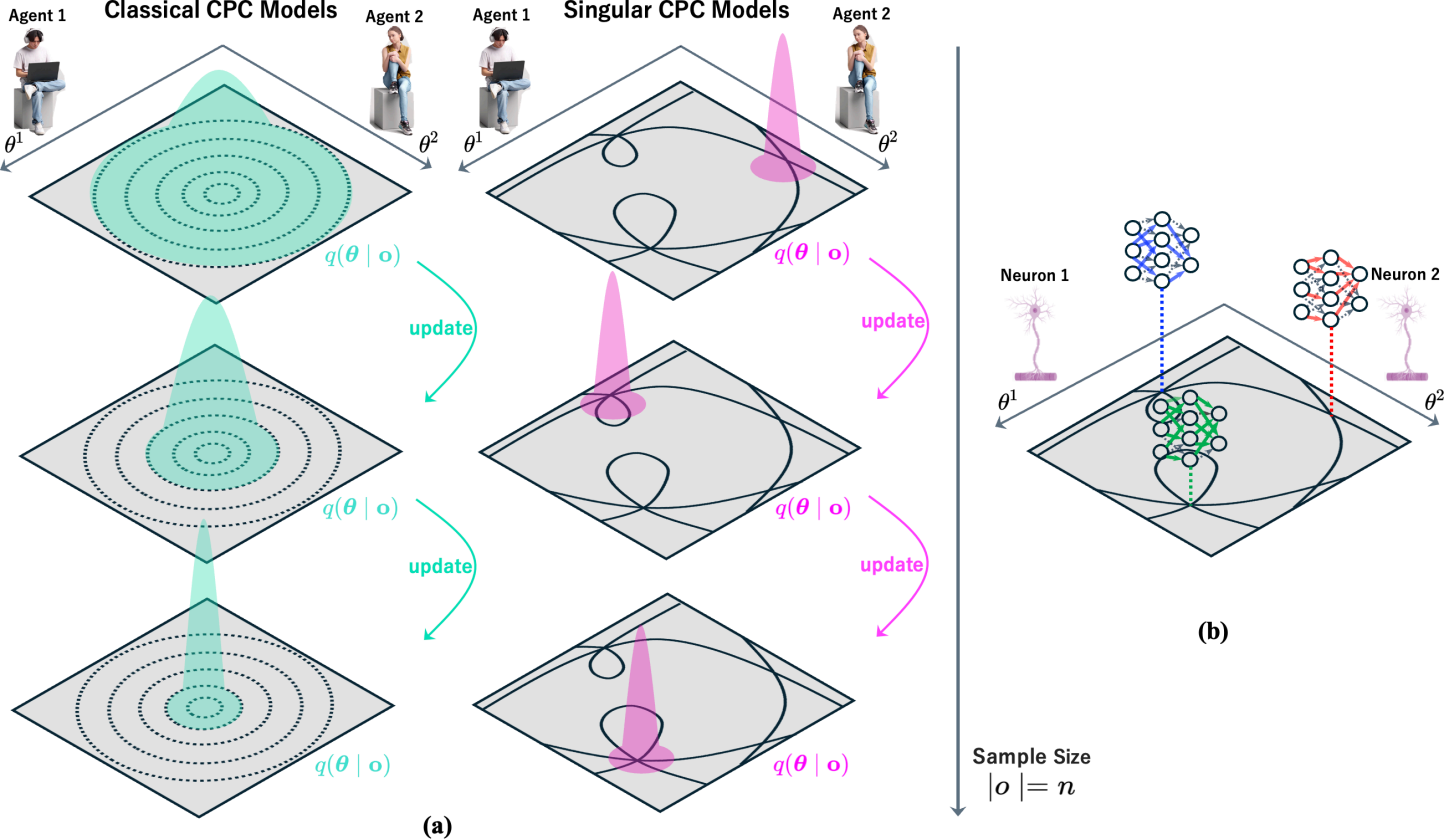
(a) Left: expected classical CPC models show gradual, continuous updates of the posterior distribution q(θ|o) as sample size increases. Right: singular CPC models demonstrate discontinuous jumps in the posterior distribution, representing paradigm shifts in scientific understanding. (b) A network representation of the singular CPC model, where scientists (Agent 1 and Agent 2) act as neurons. The connections between nodes illustrate how scientific concepts or theories interact and evolve, potentially leading to sudden structural changes in the collective knowledge network.

Science is expected to be progressive as well as objective, continually making solid improvements over past achievements. In the CPC-MS model, scientific progress is understood as the diachronic improvement of the posterior distribution through decentralized Bayesian updating. As scientific inquiry continues and more data o accumulate, the posterior distribution q(w|o) is expected to better approximate the data-generating process. This can be seen as an optimization process in which probability mass is progressively concentrated around the parameters with higher likelihood (figure 4). At the same time, this is accompanied by an optimization of the model parameter $\theta ={\left\{\theta^{k}\right\}}_{k}$, which represents the scientific understanding of each individual agent. More specifically, by mediating individual’s inner representation $z_{d}^{k}$ and observation $o_{d}^{k}$ (figure 3), each $\theta^{k}$ influences how the kth agent interprets observations and forms hypotheses about their environment. By doing so, it also serves as a bridge between individual internal representations and the shared global scientific representation $w_{d}$. This perspective allows us to view Bayesian optimization as a form of scientific progress—the process through which scientists’ understanding of the world, represented by θ, improves over time.

Under this identification, the conventional Bayesian update corresponds to a gradual improvement of the posterior distribution, where the weights of neural connectivity get refined continuously and smoothly on the basis of incoming data (figure 4a, left). This mode of updating process captures the gradual scientific progress in ‘normal science’, where the scientific community as a whole concentrates on a given vicinity of the parameter space—circumscribed by one paradigm—and seeks to its refinement.

When the parameter space of the model is singular, however, the Bayesian updating process of the posterior distribution may involve jumps from one singular point to another corresponding to a local optimal solution (figure 4b, right). In singular learning theory, the concept of a singular model refers to a model whose parameter space contains singularities, as opposed to a regular model which lacks such singularities. Developed by Sumio Watanabe, this theory employs theorems from algebraic geometry to rigorously analyse the behaviour of Bayesian updating in singular models [43,44]. The key finding is that, in stark contrast to regular models, the Bayesian updating process of the posterior distribution in singular models can exhibit discontinuous jumps from one singularity to another. These jumps correspond to phase transitions between local optima and are a frequently observed phenomenon in the Bayesian updating of posterior distributions in deep learning models. Mathematically, the singularities in the parameter space of these models determine the architecture of the model itself, dictating the presence or absence of edges connecting neurons. Consequently, learning in Bayesian deep models can be interpreted as a process whereby the model architecture is updated discontinuously via Bayesian updating of the posterior distribution. This jump of the posterior distribution from one singularity to another is called a phase transition, a phenomenon frequently observed in the Bayesian updating process of the posterior distribution in deep learning models. In deep learning models, singularities in the parameter space correspond to the architecture of the model and determine the presence or absence of edges connecting one neuron to another. In other words, learning a Bayesian deep learning model can be interpreted as a process in which the architecture of the model is updated discontinuously through Bayesian updating of the posterior distribution. In the context of the CPC framework, this can be viewed as a discontinuous change in the internal representation z of individual scientists, which in turn may induce a radical shift in the distribution of the global latent variable w.

Thomas Kuhn [2] famously described scientific progress as consisting of two distinct periods: the period of ‘normal science’, in which scientists engage in puzzle-solving activities and piecemeal refinements of a given paradigm, and the period of ‘extraordinary science’, during which accumulated anomalies encourage the critical scrutiny of foundational assumptions and exploration of new theoretical possibilities, eventually leading to a scientific revolution. The CPC-MS framework offers a Bayesian model that accommodates these two periods as distinct modes of Bayesian optimization. Normal science, in this framework, is modelled by the gradual Bayesian update of the posterior distribution towards a given local optimum. During this period, scientists espouse more or less similar local representations, making proposals of external representations that deviate significantly from the shared paradigm (namely, the set of w’s constituting a local optimum) rare, and, if made, likely to be rejected. On the other hand, scientific revolutions are represented by phase transitions that leap the posterior distribution from one singularity to another in a discontinuous fashion. Such transitions induce significant reconfigurations in the collective external representations (w) held by the scientific community, such that a set of propositions that enjoyed high probabilities before the transition cease to do so afterwards.

Kuhn infamously claimed that the incommensurability between different paradigms makes the comparison and evaluation of competing scientific theories difficult, if not impossible, because the standards of evidence, methods and the very concepts used in the theories can change so drastically between paradigms. However, within the CPC framework, there is a potential resolution to this problem that acknowledges the incommensurability while still allowing for a form of rational progress. This is because a phase transition between paradigms can still be understood as an improvement in the collective posterior distribution. Since a more refined posterior distribution enhances the explanatory power for observed data and sharpens the predictive accuracy for unobserved phenomena, the transition to a new paradigm can be considered a rational and objectively superior advancement in the scientific community’s collective understanding, even if the paradigms themselves are internally incommensurable. In this way, the CPC framework reconciles Kuhn’s notion of incommensurability with the idea of scientific progress by proposing a unified model of Bayesian optimization that accommodates both the continuous and discontinuous phases of scientific change.

### 3.3. From confirmatory to generative science

Finally, the CPC-MS framework prompts us to reconsider the very nature and purpose of the scientific enterprise. The traditional realist perspective in science views its primary goal as the pursuit of true knowledge, placing strong emphasis on the confirmatory role of scientific methods to distinguish valid hypotheses from invalid ones [1,45]. In the twentieth century, statistical tests provided precisely such a universal tool for confirmation, establishing methodological standards across various scientific disciplines [46,47]. The realist picture aligns with the aforementioned image of scientists as ‘lone truth seekers’ who independently collect data, test hypotheses and publish results, the accumulation of which forms the body of scientific knowledge.

On the other hand, the CPC framework highlights the *generative* aspect of science, viewing it as a coordinated system for producing new hypotheses, research plans, actions and predictions. In the CPC-MS model, scientific knowledge is encoded as a posterior distribution q(w|o) that reflects the collective opinions within of the scientific community. The primary function of this distributional knowledge is to induce scientists to take the next step in their inquiry. There are at least two such generative aspects. First, it serves as a key reference point for individual researchers when planning their next studies or experiments. While some researchers may strive to find exceptions to hypotheses with high posterior probability, others may design experiments that prompt a reconsideration of less attended hypotheses. In this way, scientific knowledge serves to streamline research directions that produce further evidence. This leads to a continuous cycle of proposing hypotheses, conducting experiments and refining theories, much like the iterative updates in a Bayesian model.

The second and more obvious generative role of scientific knowledge is prediction: theories are used to predict unobserved phenomena. In the Bayesian framework, a prediction of new data $\tilde{o}$ on the basis of past observation o is given by the posterior predictive distribution $p(\tilde{\text{o}}|\text{z},\text{a},\text{C})q(\text{z}|\text{o},\text{a})q(w|\text{z})$:


(3.1) $$p(\tilde{\text{o}}|\text{o},\text{a},\text{C})=\int p(\tilde{\text{o}}|\text{z},\text{a},\text{C})q(\text{z}|\text{o},\text{a})q(w|\text{z})\;dwd\text{z}.$$

Given that an accurate posterior distribution $p(\tilde{\text{o}}|\text{o},\text{a},\text{C})$ leads to better predictions, both in terms of mean squared error and the Kullback–Leibler divergence between the predicted and true distributions, the scientific progress discussed above can also be seen as an improvement in predictive performance. The emphasis on prediction becomes even more apparent in light of the discussion on active inference (§2.3), where the scientific community as a whole is viewed as a collective agent aiming to minimize the predictive error.

The shift from confirmatory to generative approaches induced by the CPC framework is intrinsically linked to its social and progressive visions of science discussed above. From the generative perspective, scientific knowledge is seen as less an end than a means that promotes scientists to pursue new projects. Social exchanges and information sharing accelerate this research cycle, enabling the scientific community as a whole to effectively explore the vast hypothesis space in an efficient manner. The focus on prediction also substantiates the common intuition that science is progressive, an idea that has posed a vexing issue for the confirmatory view of science—precisely because the history of science seemingly presents us with successive scrap-and-build cycles through the refutation of past theories rather than the cumulative build-up of true propositions over time [48]. The Bayesian perspective, on the other hand, offers a clear sense of scientific progress in terms of predictive performance, for the diachronic refinement of the posterior distribution, as discussed in the previous section, directly leads to the improvement of the posterior predictive distribution shown in equation (3.1). This suggests that the aspect of science that can be said to progress, particularly before and after a paradigm shift, is not its stock of confirmed knowledge, but rather its generative capability.

In conclusion, the CPC framework provides a comprehensive view of scientific activities that highlights their social, progressive and generative nature. This stands in contrast to the traditional view, which portrays science as a predominantly individual, cumulative and confirmatory procedure. The view of science as a generative process, driven by CPC, provides a new perspective on how scientific knowledge evolves and advances. It emphasizes the importance of diversity in scientific approaches, the value of paradigm-shifting discoveries and the collective nature of scientific progress. By understanding science through this lens, we can better appreciate the complex dynamics that drive scientific advancement and potentially develop strategies to facilitate more efficient and effective knowledge generation in the scientific community.

## 4. AI and research automation

As we have repeatedly emphasized throughout previous sections, CPC-MS provides a comprehensive model of scientific activities carried out by scientists. Notably, as discussed earlier, it uses probabilistic generative models to mathematically describe these processes. This unique characteristic of comprehensive modelling and probabilistic approaches enables CPC-MS to offer profound insights into: (i) the potential impact of AI on the future landscape of scientific research; and (ii) the development of intuitive guidelines for implementing automated scientific activities.

### 4.1. Speculating AI’s impact on science for shaping the future science

AI has made remarkable progress, its influence spreading throughout society. Science is no exception to this trend; AI has become an innovative tool in the field, and its applications in science are rapidly expanding [6,49,50]. Furthermore, AI’s development is not limited to its use as a mere tool; scientists are now exploring whether AI can do research on its own as a scientist [51,52]. They are trying to figure out if AI can come up with its own research questions, plan and run experiments and make sense of the results. Research has also increasingly explored the use of AI for generating hypotheses and producing survey papers [53–55]. The possibility of AI acting as an autonomous scientist points towards a future novel scientific community where both human and AI scientists coexist and contribute to scientific endeavours [56,57].

Given this rapid advancement of AI for science and its potential to revolutionize scientific practices, there is growing interest in how AI is affecting science. One of the most evident impacts of AI in research is its ability to dramatically enhance productivity through automation. AI can process an enormous volume of scientific literature that humans could never read, perform computations at speeds far beyond human capability, operate continuously without rest and be easily replicated, all of which contribute to significantly accelerating research progress. In the terminology of CPC-MS, this translates into an unprecedented increase in the sampling of observations $o_{d}^{k}$ and hypotheses $z_{d}^{k}$, thereby facilitating a more efficient mode of scientific process.

Another perspective is to consider the impact of AI entering the scientific community as scientists. The main feature of CPC-MS is its proposal of a model of science as a CPC activity involving multiple agents. This characteristic allows us to discuss important aspects of how AI might change the nature of science. Specifically, by modelling the scientific community as a hybrid system composed of fundamentally different agents—AI and humans—CPC-MS enables us to explore how this transformation might impact science or even alter the very nature of scientific inquiry.

For instance, the entry of AI into the scientific community might reduce the overall bias of the community. Humans are constrained by biological and cognitive limitations that restrict what they can understand, and their cognition is distorted by cognitive biases and social pressures. In the CPC framework, $\theta^{k}$ for each agent k represents these biases and hence their world models, which in turn affect the generation of $o_{d}^{k}$ and $z_{d}^{k}$. Since bias is prevalent in human agents, its effect on the global scientific representations $w_{d}$ can be proliferated through collective inference in human-only scientific community. Such bias would lead to skewed or biased inferences, the ‘blind spot’ theory and slower convergence, potentially degrading the social objectivity of science, as we have discussed in §3.1. It could also introduce noise and bias in observations or violate the independent and identically distributed (i.i.d.) condition, disturbing asymptotic improvement (§3.2).

On the other hand, AI would probably have a different $\theta^{k}$ than humans’, suggesting the possibility of generating different $o_{d}^{k}$ and $z_{d}^{k}$. Thus, the entry of AI agents into the scientific community might enable the generation of $w_{d}$ that reflects more diverse aspects of target of study d. In other words, the involvement of AI in conducting science suggests that it could introduce greater diversity, which is key for social objectivity in science as discussed in §3.1, into the process of scientific knowledge production. Therefore, AI potentially alleviates some of the limitations currently faced by human science, leading to potentially better scientific outcomes.

Simultaneously, the integration of AI also raises concerns about the shared common ground among social members, which is a prerequisite for objectivity as discussed in §3.1. CPC-MS emphasizes the role of consensus formation through communication among agents in the creation of scientific knowledge. From this perspective, science involving AI can be described as a hybrid system composed of entirely different agents—AI and humans. This bears structural similarities to the AI alignment problem [58]. In this sense, the CPC-MS framework naturally frames the challenges of AI in science as a specific example of the broader AI alignment issue.

In current science, communication between agents works well because they are all humans who share similar biological bodies and language. However, AI lacks this commonality with us, potentially raising the issue of communication barrier. This suggests that the premise of science as distributed PC, which we explained in §3.1, could collapse with the participation of AI as a scientist in the scientific community.

Moreover, a significant increase in the differences between agents could make global scientific representation production inefficient too. In a two-agent MHNG with agents k and l, for example, agent l updates $w_{d}$ by either accepting or rejecting proposals from agent k. If agent k and agent l are an AI and a human, respectively, their internal scientific representations $z_{d}^{k}$ and $z_{d}^{l}$ and then the distributions they follow are expected to be significantly different. Consequently, the generated $w_{d}^{k}$ and $w_{d}^{l}$ will also be very different. Therefore, if the original $w_{d}$ is $w_{d}^{l}$ and the proposal is $w_{d}^{k}$ , the acceptance rate is expected to be quite low (e.g. in MHNG, $w_{d}^{k}$ is accepted with probability $\text{min}(1,\frac{P(z_{d}^{k}|\theta^{k},w_{d}^{l})}{P(z_{d}^{k}|\theta^{k},w_{d}^{k})})$, and when $w_{d}^{k}$ and $w_{d}^{l}$ differ significantly, this probability is likely to be small). This could lead to poor convergence efficiency.

As the diversity and heterogeneity among agents increase, the common ground they share tends to decrease. This presents a fundamental trade-off: while greater diversity can enhance social objectivity in science, it also risks undermining the shared basis necessary for effective communication and collaboration. To maximize the potential of AI in expanding new scientific possibilities, it is thus crucial to proactively develop and establish this common ground among diverse constituents, in anticipation of AI scientists’ future participation. This involves creating a new scientific framework that maintains the benefits of heterogeneity while ensuring effective collaboration between human and AI agents.

So far, we have focused on the differences between AI and humans. However, it has also been pointed out that AI and humans share similar biases [59,60], and the internal representations of AI may be similar to each other [61]. In this case, the entry of AI into scientific research may, rather than mitigating biases, reinforce existing human biases and further steer science in that direction. While it remains an open question how AI scientists resemble human scientists and how this resemblance influences the trajectory of scientific inquiry, CPC-MS provides a framework for discussing the impact of such agents.

Like this, we can see that the integration of AI scientists into the human scientific community has the potential to significantly impact even the fundamental premises of science. Therefore, the emergence of hybrid system of AI and human scientist is creating opportunities to debate and reshape the very nature of science itself, moving beyond simply considering their impact on traditional scientific practices. Humans might have only explored a limited portion of the possible space of science [62], and it has been pointed out that the current scientific system faces many problems. The rise of AI offers us a chance to do more than just passively observe how science is changing. Instead, we can take an active role in reimagining and reshaping science itself.

### 4.2. Guideline for implementing automated total science activity

CPC-MS has the potential to provide guidelines for implementing and automating the entire scientific process of a group of scientists as a machine learning model. This could bring a new perspective to the efforts of automating science, which have traditionally focused on automating specific tasks within an individual scientist’s research workflow.

The pursuit of automating scientific practices by machines dates back to the early days of computing, with pioneering systems like DENDRAL [63] and BACON [64]. Subsequently, as exemplified by the term, fourth paradigm of science, the advancement and proliferation of computational capabilities led to the automation of certain tasks within scientific activities [65]. The 2000s saw machine learning techniques like Bayesian optimization applied to scientific processes. However, it was the deep learning revolution of the 2010s that truly accelerated the integration of AI into science, a field now known as AI for science [6,49,50]. This integration has enabled discoveries previously unsolved by humans, with AlphaFold [66] serving as a prime example.

While these activities have brought tremendous progress to humanity, they have largely been limited to the automation and substitution of a specific task in a scientific process. Current research involves activities like formulating questions, conducting experiments and writing papers. However, attempts to fully automate an entire research cycle, encompassing all of these activities from start to finish, remain few and far between.

There are few examples of attempts to automate an entire research cycle, with systems like Adam [67] and Eve [68] being notable exceptions. Adam is a closed-loop automation system that combines traditional symbolic AI with robotics to automatically generate hypotheses, verify them and modify them based on feedback. More recently, groundbreaking research has proposed an AI system capable of conducting machine learning research in a highly automated, end-to-end manner, handling a wide range of tasks from initial idea generation to paper writing [52].

However, even these attempts are specialized, focusing on automating specific areas of scientific research rather than achieving a general automation of science. Furthermore, the automation achieved so far typically replicates the research process of a single scientist. The AI scientist by Lu *et al*. [52] represents a significant step forward in automating the social aspects of scientific work, as it not only automates paper writing but also the peer-review process. However, even this study still falls short of automating the entire scientific activities.

As this article emphasizes, science is inherently a collaborative effort. It is common for multiple researchers to work together to understand a single phenomenon, and peer review—the process by which other researchers validate research outcomes—is a crucial aspect of scientific activity. The existing closed-loop research automation systems have not yet managed to incorporate these social aspects, leaving the automation of the entire scientific process, including its collaborative nature, still unrealized.

To achieve this goal, we need a model that captures both the social dimensions of science and the entire process from discovery to knowledge representation and communication by scientists, without being too tied to specific scientific practices. Moreover, to actually automate this model in practice, it needs to be implementable, which means it must be expressible mathematically. CPC-MS offers such a model of science. In essence, CPC-MS could serve as a foundation or starting point for developing a system capable of automatically executing the full spectrum of scientific activities performed by a scientific community.

CPC-MS takes a unique approach by modelling science as a probabilistic generative process. This perspective allows us to view the whole range of scientific activities through the lens of statistical machine learning, enabling us to potentially implement these activities as a machine learning model. Furthermore, by framing science in terms of generative models, CPC-MS can easily incorporate the latest developments in generative AI—a field that has been making waves across society in recent years. To conclude, CPC-MS offers a novel framework for understanding and advancing the future direction of research automation.

## 5. Future work

### 5.1. Network structure in scientific communities

The CPC framework can be extended by considering the network structure of multiple agents. Interactions between multiple agents with the CPC framework describe biases and effects in scientific communities. Global scientific representations are often generated by a small scientific community with a few dozen members. Demographics of such small community may bias or influence scientific activities such as paper citation [69], peer review [70] and recruiting [71]. For example, gendered citation patterns are known in contemporary physics [69]. This pattern can be described as inferences from a smaller number of agents. A small number of agents easily biases collective decision making.

Another bias can come from whether researchers work with famous or non-famous researchers, notably the Matthew effect [72,73]. The difference between famous and less famous researchers may influence range of broadcasting global representations. Less famous researcher may not be able to broadcast global scientific representations to other members effectively while famous researcher can do. A similar pattern also appears in famous and less famous research institutes [74]. Academic success may also be influenced by the structure of social network. For example, diverse intellectual synthesis between mentors and mentees influences success in academic careers [71].

It is also interesting to discuss the influence of scientific communities on the growth of scientific concepts. Scientific concepts are developed within scientific communities composed of many researchers, so the dynamics of these concepts reflect the decision-making processes of these communities. Scientific concepts evolve by filling knowledge gaps, and scientific discoveries are often accompanied by the awarding of the Nobel Prize as an indicator of recognition by the scientific community [75]. Scientific consensus, measured by the frequency of consensus-related words such as ‘agreement’ in academic papers, is associated with the dynamics of the network structure of scientific concepts [76]. Since the CPC framework was developed to explain symbol emergence in humans, it should also be capable of describing how scientific concepts are developed, accepted or rejected by scientific communities through the interactions of multiple agents.

It is worth mentioning the relationship with science of science (SciSci) [5]. SciSci is an emergent interdisciplinary field to uncover mechanisms of doing science using computational methods. Geographical and temporal interactions between scientists are targets of SciSci in addition to conventional citation and co-authorship analyses. The CPC framework is based on interactions between multiple agents and concurs with SciSci from the perspective of analysing network structure.

Furthermore, SciSci also aims to influence the network structure of agents and scientific activities. One solution is to create open datasets and softwares visualizing biases in scientific activities. Some studies contribute to policy making in scientific commercialization [77] and public funding [78,79]. Ohniwa *et al*. showed that diverse and smaller grants are more effective than centralized and larger grants to generate new fields and promote technology transfer in Japan public funding [79]. Such evidence may theoretically support effectiveness of decentralized communities, so-called decentralized autonomous organizations, to support science projects [80]. Recently, there has been a growing momentum around the movement known as decentralized science (DeSci). DeSci are practices of science or related activities, which often are designed with decentralized mechanisms and technologies such as blockchain. In this regard, the CPC theory can be extended by theoretical understanding of social biases and computational frameworks to analyse new forms of science management models including DeSci. Further studies of social network will contribute to the extension of the CPC framework.

### 5.2. Simulation study of collective predictive coding science model

CPC provides a computational model of science. Therefore, by executing this model on a computer, it is possible to simulate how the scientific community produces knowledge. The advantage of being able to simulate science is that it allows us to conduct pseudo-experiments on science, which is a social activity where actual experiments are not possible. This enables us to discuss how various factors could potentially influence the scientific community and in what ways. For instance, changing the distribution that $\theta^{k}$ follows for each k may allow us to study how the diversity among scientists actually influences scientific activity (§§3.1 and 4.1). Moreover, if we limit the communication to agents within the same cluster c and restrict inter-cluster communication, we may be able to reveal the impact of clustering on the production of global scientific representations in scientific communities, as discussed above. Additionally, introducing malicious players who make false reports, for example, we may introduce players with an irrational acceptance ratio of $w_{d}$ or players that randomly suggest $w_{d}^{k}$ independent of $z_{d}^{k}$. Alternatively, we may also introduce agents that excessively prioritize individual epistemic value or pragmatic values (§2.3) and how these agents affect scientific integrity.

### 5.3. Non-stationarity

In the current CPC-MS, for simplicity, it is implicitly assumed that the distribution that $w_{d}$ follows is stationary. However, in reality, the distribution that $w_{d}$ follows may change over time. For example, in fields where claims are difficult to verify and established theories can change significantly when new evidence emerges, the distribution of $w_{d}$ is expected to be non-stationary. Extending CPC-MS to naturally model such non-stationary distributions of $w_{d}$ is one potential direction for expanding the framework.

### 5.4. Causal inference in scientific activities

The current CPC-MS framework primarily focuses on correlational patterns between observations and internal/external representations within scientific activities. However, a significant aspect of scientific inquiry involves establishing causal relationships rather than mere correlations—scientists often aim to determine what causes what, not just what predicts what. While our current formulation captures the predictive aspect of science through the Bayesian inference framework, it does not explicitly address how causal inferences are made or how interventional data (as opposed to observational data) is integrated into the scientific process. Judea Pearl’s framework of causal inference distinguishes between statistical predictions and causal statements, where the latter requires intervention in the system being studied [81]. Scientists make causal statements by taking action—by manipulating variables and observing the effects—going beyond inferences about language or symbols. Future extensions of CPC-MS could incorporate formal causal modelling tools such as causal Bayesian networks, do-calculus or potential outcomes framework to explicitly represent how scientists form and test causal hypotheses [82–84]. This extension would bridge the gap between the current predictive framework and the causal nature of scientific explanation, potentially providing insights into how collective scientific activities establish causal knowledge despite individual limitations in experimentation and observation.

### 5.5. Incorporating external players

In the current framework, only scientists are considered agents, but in actual science, multiple non-scientist players are essential. These include funders, influencers of scientific direction and educators. The current CPC-MS framework does not explicitly model these players, limiting discussions of their influence. Incorporating non-scientist players could extend the model. For example, funders influence research themes, indirectly corresponding to selecting certain object d to be studied from this world. This decision making is closely related to how $w_{d}$ is used and what is required as $w_{d}$ for a society [78]. The current framework models the inference of $w_{d}$ of target of study but does not address how the target is selected or how $w_{d}$ is used. Modelling such target selection process, as mentioned above, could provide deeper insights into the effect of the society to science.

### 5.6. Utilities and values

When selecting research topics, the value to society and individuals becomes a crucial factor driving research activities. At first glance, the generative model of CPC (figure 3) may appear to focus solely on the encoding of observational information, without incorporating elements such as individual values or rewards. However, the PGM of CPC can be naturally extended to include decision making based on rewards and values. In many cases, decision making based on rewards and values can be reformulated as Bayesian inference of action sequences to reach a desired state [85,86]. Building on this theory, Ebara *et al*. have proposed a multi-agent reinforcement learning algorithm that extends the MHNG based on CPC to include symbol emergence [87].

Following the formulation of active inference presented in §2.3, pragmatic value can be used to model both utilities and values.

Based on the CPC-MS framework, future challenges include discussing the selection of research topics based on values and the impact of individual agents’ (researchers’) incentives on scientific exploration as CPC.

## 6. Conclusion

The CPC-MS framework presented in this article offers a novel perspective on scientific activities, viewing them through the lens of CPC and decentralized Bayesian inference. By modelling science as a generative process carried out by a community of agents—namely, generative science—CPC-MS provides several key insights.

It formalizes the social nature of scientific knowledge production, demonstrating how individual observations and hypotheses are integrated into explicit scientific knowledge, i.e. global scientific representations, through communication and peer review. The CPC-MS framework offers a mathematical foundation for understanding scientific progress, paradigm shifts and the role of diversity in scientific communities. It bridges the gap between individual cognitive processes and collective knowledge creation in science, offering a unified view of scientific activities, from experimentation to theory development.

The CPC-MS framework aligns with and extends existing ideas in the philosophy of science, such as social objectivity and the generative nature of scientific theories. It encourages us to view science not just as a collection of facts or theories, but as a dynamic, collective cognitive process that continually refines our understanding of the world.

As we move towards an era where AI plays an increasingly significant role in scientific research, the CPC-MS framework provides a valuable tool for understanding and shaping the future of science. Additionally, the mathematical nature of CPC-MS allows us to integrate generative AI-based automated scientific research as a part of the CPC-MS framework.

Future work could focus on refining the mathematical models underlying CPC-MS, conducting empirical studies to test its validity in modelling actual scientific studies, and exploring its implications for science policy, research management and research ethics. Ultimately, this framework aims to contribute to a more comprehensive understanding of how scientific knowledge is created, validated and advanced through collective effort.

## Data Availability

This article has no additional data.

## Footnotes

^1^
Note that there are two types of interpretation of the review process. One is that two researchers, the kth and the $k^{\prime }$th agents, have different observations $o^{k}\neq o^{k^{\prime }}$, and the reviewer $k^{\prime }$ judges the paper w written by the kth agent, i.e. a sample from $p(w|o^{k})$, by using $q(z^{k^{\prime }}|o^{k^{\prime }})$. However, in this case, if there is a significant difference between the distributions $p(o^{k})$ and $p(o^{k^{\prime }})$, it may result in an unfairly low acceptance ratio. The other interpretation is that two researchers share the same data $o^{k}=o^{k^{\prime }}$, i.e. the data is shared publicly. In this case, the reviewer uses their knowledge, i.e. the global parameter of $p(z^{k^{\prime }}|w)$ and $p(z^{k^{\prime }}|o^{k^{\prime }}=o^{k})$, without being affected by the difference in data distributions.

## Appendix A. Nomenclature

Table 2 shows a list of symbols used in this article and their descriptions.

**Table 2.** Nomenclature of CPC-MS.

symbol	description
$w_{d}$	external representation for the dth target
$z_{d}^{k}$	internal representation of the kth agent for the dth target
$o_{d}^{k}$	observation of the kth agent for the dth target
d	index for target objects or phenomena being studied
k	index for individual agents (e.g. scientists)
$\theta^{k}$	local parameters for the kth agent (e.g. learned internal models)
K	total number of agents
D	total number of objects
$p(w_{d})$	prior distribution of external representations for the dth object
$p(o_{d}^{k}|z_{d}^{k})$	likelihood of observations given internal representations for the kth agent and dth object
$p(z_{d}^{k}|w_{d})$	prior distribution of internal representations given external representations
$q(w_{d}|\{z_{d}^{k}{\}}_{k})$	approximate posterior distribution of external given internal representations
$q(z_{d}^{k}|o_{d}^{k})$	approximate posterior distribution of internal representations given observations
$p(w_{d}|\{o_{d}^{k}{\}}_{k})$	true posterior distribution of external representations given observations
$q(w_{d}|\{o_{d}^{k}{\}}_{k})$	approximated posterior distribution of external representations given observations
$a^{k}$	actions taken by the kth agent (e.g. conducting experiments)
$C^{k}$	rewards taken by the kth agent (e.g. budget for the experiments)
F	variational free energy
G(a)	expected free energy for future actions (bold a represents actions of all agents)

## Appendix B. Metropolis–Hastings naming game and its extensions

The original idea of MHNG was introduced by [88], and extended and generalized by [27] to involve representation learning.

The MHNG defined in [27] consists of the following steps.

(1) **Perception**. Speaker and listener agents (*Sp* and *Li*) observe the dth object, obtain $o_{d}^{Sp}$, and $o_{d}^{Li}$ infers their internal representation $z_{d}^{Sp}$ and $z_{d}^{Li}$, respectively.
(2) **MH communication**. Speaker tells the name $w_{d}^{Sp}$ of the dth object by sampling it from $P(w_{d}|z_{d}^{Sp},\phi^{Sp})$. The listener determines if it accepts the naming with probability $r=\text{min}\left(1,\frac{P(z_{d}^{Li}|\phi^{Li},w_{d}^{Sp})}{P(z_{d}^{Li}|\phi^{Li},w_{d}^{Li})}\right)$.
(3) **Learning**. After MH communication was performed for every object, the listener updates its global parameters $\theta^{Li}$ and $\phi^{Li}$.
(4) **Turn-taking**. The speaker and listener alternate their roles and go back to (1).

They showed that the MHNG is a Metropolis–Hastings sampler of $p(w,z^{1},z^{2},\theta^{1},\theta^{2},\phi^{1},\phi^{2}|o^{1},o^{2})$*,* where $\theta^{k}$ and $\phi^{k}$ are global parameters of the generative model corresponding to the kth agent. As a result, the MHNG can perform fully decentralized approximate Bayesian inference of w. For more detail, please refer to the original paper [27]. Taniguchi *et al*. validated the MHNG on a PGM called Inter-GMM+VAE, modelling a two-agent symbol emergence system [9,10] using Gaussian mixture model (GMM) for $p(z^{k}|w)p(w)$ and variational autoencoder (VAE) for $p(o^{k}|z^{k})p(z^{k})$ and combining them using the Neuro-SERKET framework [89]. While initial MHNG studies focused on two-agent systems, Inukai *et al*. [28] extended this to N-agent conditions by introducing recursive communication structures.

Although these theories originally define signs categorically, the MHNG framework naturally allows for broader types of signs, including compositional word sequences [90] and continuous signs like voice pitch [91]. Saito *et al*. [92] modelled the emergence of compositional signs from continuous time-series signals.

To validate the CPC hypothesis and MHNG’s validity in explaining human symbol emergence behaviours, Okumura *et al*. [93] conducted experimental semiotic studies and showed that MHNG’s sign acceptance rate effectively predicts human behaviour in joint-attention naming games, bridging theoretical models with empirical evidence.
